# Dexmedetomidine-Based Anesthesia for Pediatric Adenotonsillectomy: A Retrospective Observational Case Series

**DOI:** 10.7759/cureus.86649

**Published:** 2025-06-24

**Authors:** Abdulrazzg A Mohammad, Abdullah Almehari, Abdulaziz F Alajmi, Muawad Rayan, Ahmed Haroun M Mahmoud

**Affiliations:** 1 Department of Pediatric Anesthesia, King Abdullah Specialized Children Hospital, Ministry of the National Guard - Health Affairs, Riyadh, SAU; 2 Research and Development, King Abdullah International Medical Research Center, Riyadh, SAU; 3 Medical Education, College of Medicine, King Saud bin Abdulaziz University for Health Sciences, Riyadh, SAU; 4 Medicine, King Saud bin Abdulaziz University for Health Sciences College of Medicine, Riyadh, SAU

**Keywords:** opioids, pediatric adenotonsillectomy, pediatric obstructive sleep apnea, postoperative pain control, dexmedetomidine

## Abstract

Background

Tonsillectomy is a surgical procedure primarily aimed at the removal of the tonsils, which are lymphoid tissues located at the back of the throat. This procedure is commonly indicated for conditions such as recurrent tonsillitis, chronic tonsillitis, or obstructive sleep-disordered breathing resulting from enlarged tonsils, which is often conducted under general anesthesia. The purpose of this research is to assess the safety and efficacy of dexmedetomidine-based anesthesia with minimal opioid supplementation in children undergoing adenotonsillectomy.

Methods

We studied pediatric patients who underwent adenotonsillectomy as a day case from March 2024 to September 2024. We reviewed patients who received dexmedetomidine as the primary analgesic agent, with minimal supplemental opioid use, intravenously after standard inhalation of anesthesia and after the surgery. The dose was determined by the anesthesiologist based on age, weight, and anesthetic requirements. There was no fixed institutional protocol for dose standardization. For pain assessment, we used the Face, Legs, Activity, Cry, and Consolability (FLACC) Scale. We looked at any patient who required postoperative analgesia in the post-anesthesia care unit (PACU). We reviewed cases conducted in the pediatric operating room and recovery unit at King Abdullah Specialized Children Hospital in Riyadh, Saudi Arabia. While FLACC is validated for patients aged two months to seven years, it was used in all patients due to institutional practice. This is acknowledged as a limitation.

Results

A total of 51 pediatric patients were included in this study. The study population was divided into three age groups: Group 1 (<3 years, n = 12), Group 2 (>3 to <7 years, n = 21), and Group 3 (>7 years, n = 18). This age-based stratification was used to assess whether patient age influenced postoperative outcomes such as pain scores, recovery duration, or oxygen requirements. The mean dose of dexmedetomidine (Precedex) administered intraoperatively was 0.66 mcg/kg (range: 0.16-1.4), while the mean fentanyl dose was considerably lower at 0.17 mcg/kg (range: 0.00-3.0), indicating minimal reliance on opioids. Spearman correlation analysis did not reveal any statistically significant associations between the primary independent variables (Precedex dose, fentanyl dose, surgery duration, and age group) and the postoperative outcomes of interest.

Conclusion

This retrospective case series suggests that dexmedetomidine may offer a feasible opioid-sparing approach to postoperative analgesia in pediatric adenotonsillectomy. However, due to the small sample size and lack of a control group, conclusions about efficacy should be interpreted with caution.

## Introduction

Tonsillectomy is one of the most common ambulatory procedures performed worldwide. According to the 2017 National Health State Report, approximately 289,000 tonsillectomies were performed annually in the United States in children under 15 years of age [1]. Locally, at King Abdullah Specialized Children Hospital in Riyadh, Saudi Arabia, more than 2,000 adeno-tonsillectomies were performed during the COVID-19 pandemic (2019-2021). Recurrent throat infections and obstructive sleep-disordered breathing are the most common indications for tonsillectomy, as outlined by the American Academy of Otolaryngology [2]. This procedure is typically conducted under general anesthesia on an outpatient basis, allowing most patients to be discharged the same day. However, children with obstructive sleep apnea (OSA) are at higher risk of postoperative respiratory complications, particularly when opioids are used for pain management. OSA, characterized by partial or total airway obstruction during sleep, can result in hypoxemia, hypercarbia, fragmented sleep, and behavioral changes [3]. Data from malpractice claims highlight the dangers of opioid use in these patients.

Sleep apnea was implicated in 17 fatal and 15 non-fatal malpractice cases following tonsillectomy, and evidence suggests that at least 16 children could have been rescued with proper postoperative monitoring [4,5]. In addition, pharmacogenetic studies have demonstrated that children with increased CYP2D6 activity - ultra-rapid or extensive metabolizers -are at heightened risk for codeine-related adverse effects, such as sedation and respiratory depression [6]. This has led to FDA warnings against codeine use in children undergoing tonsillectomy, prompting a shift toward opioid-free pain management strategies [7]. Dexmedetomidine, a selective alpha-2 adrenergic agonist, offers sedative, anxiolytic, and analgesic properties without causing respiratory depression, making it an appealing alternative to opioids. Although initially approved by the FDA for adult use, recent reviews have indicated no new pediatric safety concerns [8,9]. Reported side effects, such as hypotension and bradycardia, occur in fewer than 2% of patients and are generally manageable [9,10]. While dexmedetomidine is not approved for pediatric use by regulatory authorities, multiple recent reviews have supported its off-label use in pediatric anesthesia and critical care [11]. 

The purpose of this research is to describe outcomes associated with the intraoperative use of dexmedetomidine with minimal opioid supplementation in pediatric tonsillectomy cases.

## Materials and methods

After acquiring the approval from the Institutional Ethics Committee of King Abdullah International Medical Research Center (KAIMRC) (approval no. NRR24/031/7), we studied pediatric patients who underwent adenotonsillectomy as a day case surgery. We looked at patients who received dexmedetomidine, with minimal opioids, intravenously after standard inhalation of anesthesia and after the surgery. The dose was determined by the anesthesiologist based on age, weight, and anesthetic requirements. There was no fixed institutional protocol for dose standardization. We looked at any patient who required postoperative analgesia in the post-anesthesia care unit (PACU). We reviewed cases that were conducted in the pediatric operating room and recovery unit at King Abdullah Specialized Children Hospital in Riyadh, Saudi Arabia. 

Primary objective

This study aimed to describe outcomes associated with the intraoperative use of dexmedetomidine with minimal opioid supplementation in pediatric tonsillectomy cases.

Secondary objectives

The study also aims to investigate potential correlations among several factors within the PACU. These factors include the length of stay in the PACU, the proportion of patients requiring opioids while in the PACU, the necessity for oxygen supplementation in the PACU, and the occurrence of any complications during the PACU stay. By analyzing these variables, the study seeks to understand potential relationships and implications for patient care and recovery in the postoperative setting.

Study design

A retrospective observational case series was chosen for the study, involving a sample size of 51 patients who met the inclusion and exclusion criteria.

Inclusion and exclusion criteria

The inclusion criteria of the study were any patient aged two to 14 years old who underwent adenotonsillectomy as day surgery and patients with a history of snoring or sleep-disordered breathing were also included in the study. As for the exclusion criteria, it was any patient aged less than two years old or above 14 years old and patients who were already admitted to the hospital before surgery for another reason.

Sample size

After following the inclusion and exclusion criteria, we studied a total of 51 patients.

Sampling technique

Retrospective chart reviews for all adenotonsillectomy patients were collected at King Abdullah Specialized Children Hospital from March 2024 to September 2024.

Data analysis

Descriptive statistics were used to summarize patient characteristics and intraoperative variables. Continuous variables such as Precedex dose, fentanyl dose, surgery duration, anesthesia duration, PACU stay duration, oxygen application duration in the PACU, and time to eye opening post-surgery were expressed as mean, minimum, maximum, and range. Patients were stratified into three age groups (Group 1: ≤3 years, Group 2: >3 to <7 years, Group 3: ≥7 years). Group-wise comparisons of continuous outcomes were performed using the Kruskal-Wallis test, as data did not follow a normal distribution. Spearman correlation coefficients were calculated to evaluate the relationship between independent variables (Precedex dose, fentanyl dose, surgery duration, and age group) and dependent outcomes (time to eye opening, PACU duration, oxygen application duration, and pain scores in PACU). To assess the association between categorical variables, such as OSA presence and PACU pain scores, a chi-square test was applied. A p-value of <0.05 was considered statistically significant. All statistical analyses were performed using Python (pandas, SciPy). All statistical analyses were conducted using Python (version 3.11.8, with libraries including pandas, SciPy.stats). Descriptive statistics were used to summarize continuous variables as mean, minimum, maximum, and range. Group comparisons across age categories were performed using the Kruskal-Wallis test. Correlations between continuous variables were assessed using Spearman’s correlation coefficients. Associations between categorical variables were evaluated using the chi-square test. A p-value of <0.05 was considered statistically significant.

## Results

A total of 51 pediatric patients were included in this study. Females represented 21 (41.18%) of the patients, while males accounted for 30 (58.82%). The mean dexmedetomidine dose was 0.66 mcg/kg (range: 0.16-1.4 mcg/kg). Nine patients (17.6%) received supplemental fentanyl with a mean dose of 0.17 mcg/kg among those who received it (range: 0.00-3.0 mcg/kg). The average surgery duration was 18.41 minutes, with anesthesia lasting a mean of 31.88 minutes. Postoperative recovery parameters showed moderate variation: patients stayed in the PACU for an average of 50.39 minutes, received oxygen supplementation for a mean of 17.04 minutes, and regained eye opening at a mean of 25.76 minutes postoperatively (Table 1).

**Table 1:** Summary of the descriptive statistics for key intraoperative and postoperative variables. PACU*: post-anesthesia care unit

Variable	Mean	Min	Max	Range
Precedex dose (mcg/kg)	0.66	0.16	1.4	1.24
Fentanyl dose (mcg/kg)	0.17	0.00	3.0	3.00
Surgery duration (min)	18.41	6	42	36.00
Anesthesia duration (min)	31.88	12	65	53.00
PACU* stay duration (min)	50.39	27	142	115.00
O2 application (min)	17.04	2	34	32.00
Time to eye opening (min)	25.76	2	51	49.00
Pain score	0.549	0	8	0-8

**Figure 1:**
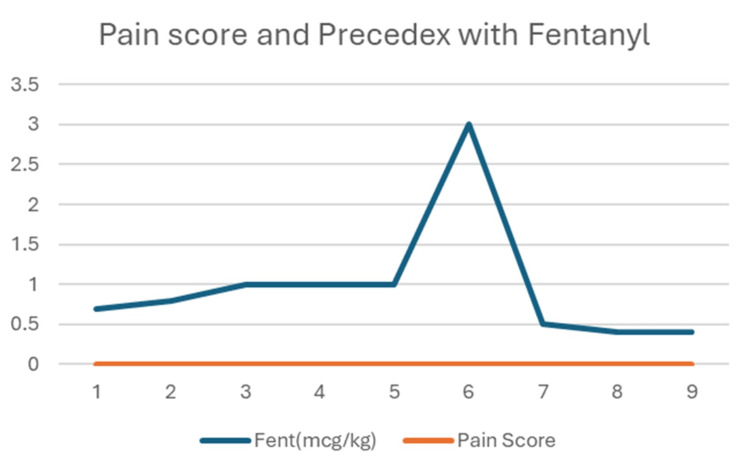
Graph demonstrating a general trend of pain scores in patients receiving dexmedetomidine and those receiving dexmedetomidine and fentanyl

Figure 1 shows a line graph plotting: fentanyl dose (mcg/kg) shown by the blue line and pain score shown by the orange line across a smaller subset of patients (x-axis: patients who received both Precedex and fentanyl). The doses start around 0.6-1 mcg/kg, rise sharply at patient 6 to ~3 mcg/kg, and then drop again. This indicates variation in fentanyl dosing, possibly tailored to surgical or patient factors. Pain score (orange line) remains consistently zero across all nine patients. This implies the effectiveness of analgesia in all patients who received both Precedex and fentanyl.

**Figure 2:**
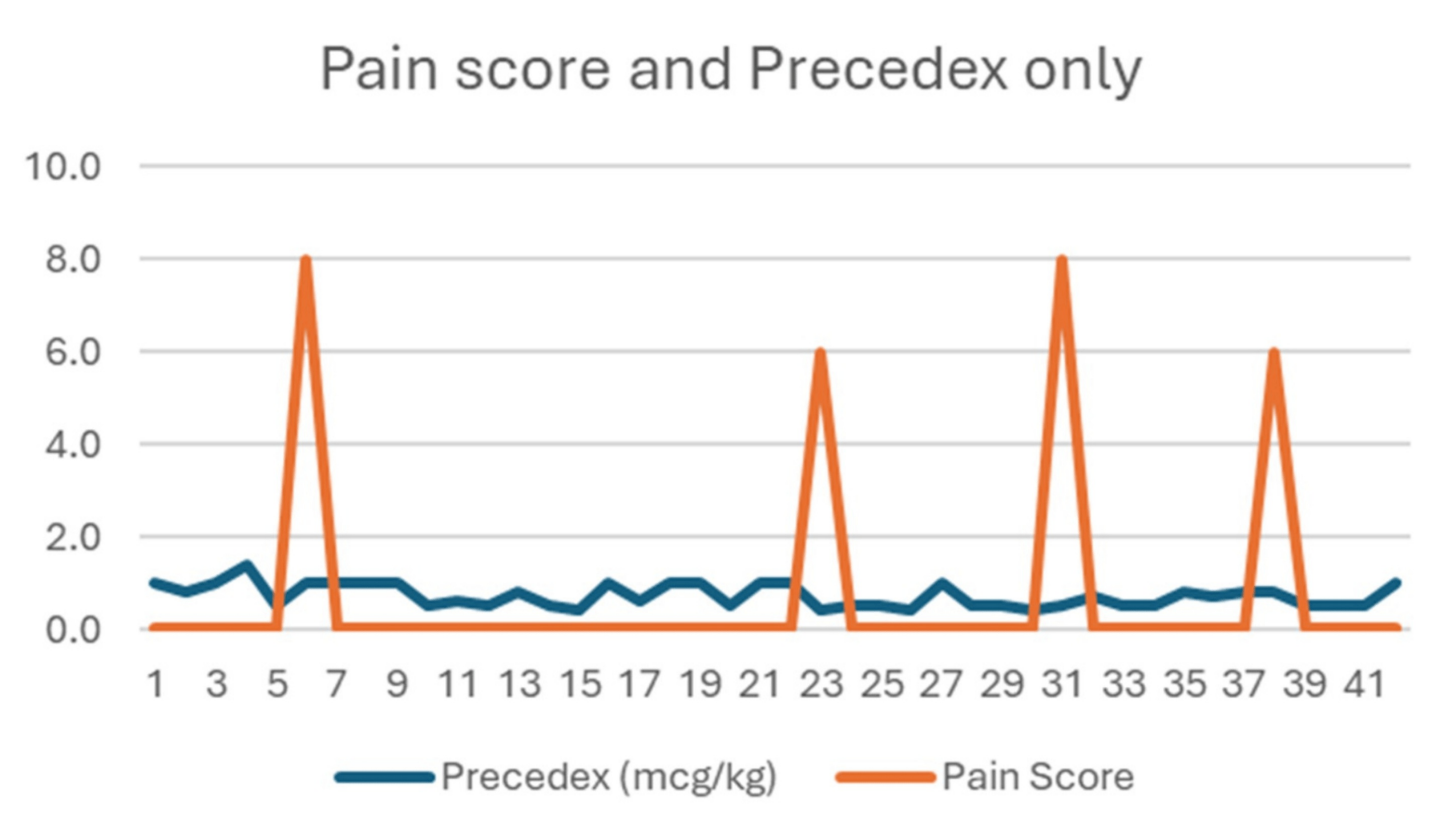
Distribution of pain scores by Precedex only.

The combination of Precedex with fentanyl appears to result in excellent pain control in this group. Even patients who received lower fentanyl doses (0.5-1 mcg/kg) had no recorded pain, suggesting a possible synergistic effect with dexmedetomidine. The absence of pain spikes seen in Figure 2 supports the idea that adding fentanyl may improve consistency in pain control.

The study population was divided into three age groups: Group 1 (<3 years, n = 12), Group 2 (>3 to <7 years, n = 21), and Group 3 (>7 years, n = 18). Group 2 represented the largest proportion of patients. This age-based stratification was used to assess whether patient age influenced postoperative outcomes such as pain scores, recovery duration, or oxygen requirements (Table 2).

**Table 2:** Age group distribution y*: years

Age group	Number
Group 1 (<3 y*)	12
Group 2 ( >3 - <7 y*)	21
Group 3 (>7 y*)	18

Spearman correlation analysis did not reveal any statistically significant associations between the primary independent variables (Precedex dose, fentanyl dose, surgery duration, and age group) and the postoperative outcomes of interest. Notably, there was no significant correlation between Precedex dose and time to eye opening (ρ = 0.049, p = 0.735), PACU stay duration (ρ = -0.023, p = 0.870), oxygen duration (ρ = 0.003, p = 0.984), or pain scores (ρ = 0.001, p = 0.993). Fentanyl dose and surgery duration similarly showed no meaningful correlations with any postoperative measures. Although the age group demonstrated a borderline association with pain scores (ρ = 0.250, p = 0.076), this did not reach statistical significance (Table 3).

**Table 3:** Spearman correlation analysis revealed no statistically significant associations (p > 0.05) between any independent variable and postoperative outcomes. PACU*: post-anesthesia care unit

Independent variable	Dependent variable	Correlation (p)	p-value
Precedex dose	Time to eye opening	0.049	0.735
	PACU* stay duration	-0.023	0.870
	Oxygen duration in PACU*	0.003	0.984
	Pain score	0.001	0.993
Fentanyl dose	Time to eye opening	0.098	0.495
	PACU* stay duration	0.160	0.263
	Oxygen duration in PACU*	0.016	0.914
	Pain score	-0.134	0.348
Surgery duration	Time to eye opening	0.088	0.537
	PACU* stay duration	0.138	0.334
	Oxygen duration in PACU*	-0.159	0.266
	Pain score	0.112	0.433
Age group	Time to eye opening	0.167	0.241
	PACU* stay duration	0.072	0.618
	Oxygen duration in PACU*	-0.032	0.821
	Pain score	0.250	0.076

Further subgroup analysis using the Kruskal-Wallis test confirmed that there were no statistically significant differences in postoperative outcomes across the three age groups. Time to eye opening (H = 1.50, p = 0.473), PACU stay duration (H = 0.26, p = 0.876), oxygen duration in PACU (H = 0.69, p = 0.710), and pain scores (H = 3.30, p = 0.192) were not significantly affected by age category (Table 4).

**Table 4:** The Kruskal-Wallis test showed no statistically significant differences across age groups for any of the dependent variables. PACU*: post-anesthesia care unit

Dependent variable	H-statistic	p-value
Time to eye opening	1.50	0.473
PACU* stay duration	0.26	0.876
Oxygen duration in PACU*	0.69	0.710
Pain score	3.30	0.192

Lastly, a chi-square test was used to explore the association between the presence of OSA and reported pain scores. The results indicated no statistically significant relationship (χ² = 1.86, p = 0.394), suggesting that OSA status did not influence postoperative pain levels in this cohort (Table 5).

**Table 5:** Association between the presence of obstructive sleep apnea (OSA) and reported pain scores. OSA*: obstructive sleep apnea

Variable comparison	Chi-square statistic	p-value
OSA* presence vs. pain score	1.86	0.394

No patients experienced intraoperative or postoperative bradycardia or hypotension requiring pharmacologic intervention. PACU and anesthesia records did not report hemodynamic instability. The absence of delayed recovery further supports the clinical safety of dexmedetomidine in our patient cohort.

## Discussion

This study evaluated the efficacy and safety of dexmedetomidine-based anesthesia in pediatric patients undergoing adenotonsillectomy. However, we note that a direct comparison to traditional opioid-based regimens was not possible due to the absence of a control group. The primary outcome was postoperative pain control in the PACU, while secondary outcomes included PACU length of stay, need for supplemental oxygen, and the occurrence of complications. Our findings suggest that dexmedetomidine provides effective analgesia in the immediate postoperative period, as reflected by the relatively low pain scores across all age groups. The absence of statistically significant differences in pain scores across age groups (p = 0.192) and the lack of significant correlation between Precedex dose and pain scores (p = 0.993) suggest a consistent analgesic effect regardless of age or dose variation within the studied range. This supports existing literature highlighting dexmedetomidine’s potential as a reliable alternative to opioids for postoperative pain management in children [12]. In addition to analgesia, dexmedetomidine has demonstrated efficacy in reducing emergence delirium (ED) and agitation following tonsillectomy and adenoidectomy [13]. A recent meta-analysis concluded that dexmedetomidine significantly lowers Pediatric Anesthesia Emergence Delirium (PAED) scores and reduces the incidence of ED and emergence agitation [13].

Although our study did not specifically measure ED, clinical observation indicated calm emergence in most patients, supporting its sedative benefits. Importantly, dexmedetomidine was associated with a low incidence of respiratory complications. The need for oxygen supplementation in the PACU was limited (mean = 17.04 minutes), and no correlation was observed between Precedex dose and oxygen duration (p = 0.984), reinforcing the agent’s known respiratory safety profile [14]. Previous studies, including meta-analyses, have consistently reported that dexmedetomidine preserves respiratory function and reduces opioid-related hypoventilation risk [15,16]. The chi-square analysis showed no significant relationship between the presence of OSA and elevated pain scores (p = 0.394), suggesting that dexmedetomidine may offer consistent analgesic benefits even in higher-risk subpopulations, such as OSA patients. This observation is aligned with findings from [16]. Who reported favorable outcomes with dexmedetomidine in OSA patients undergoing adenotonsillectomy without increased respiratory compromise [16]. In addition, none of the secondary outcomes, i.e., PACU stay duration, oxygen requirement, or time to eye opening, showed statistically significant differences across age groups. No meaningful correlations were found between these outcomes and intraoperative variables such as fentanyl dose or surgery duration. These findings may reflect the relatively uniform and short duration of surgery across the sample (mean = 18.41 minutes), as well as the standardized perioperative protocols in our specialized pediatric center.

The inclusion of a small proportion of patients (n = 9) who received intraoperative fentanyl (mean dose = 0.17 mcg/kg) introduces a potential confounding variable. However, the weak correlation between fentanyl dose and pain scores (p = 0.348) suggests that the overall analgesic effect can likely be attributed to dexmedetomidine. Furthermore, the literature supports that higher-dose dexmedetomidine can prolong the opioid-free interval postoperatively and reduce opioid consumption [15]. Another consideration is dexmedetomidine’s known hemodynamic effects, particularly bradycardia and hypotension, due to its α2-adrenergic activity. Although no hemodynamic instability was observed in our cohort, previous studies have highlighted the importance of careful monitoring, especially in children with underlying cardiovascular vulnerabilities [17]. Recent evidence also suggests that combining dexmedetomidine with local anesthetics such as ropivacaine may further enhance analgesic outcomes [18]. The study demonstrated that local infiltration with dexmedetomidine and ropivacaine significantly reduced postoperative pain in tonsillectomy patients [18]. Future studies could explore the benefits of such multimodal approaches.

Despite these encouraging findings, our study has several limitations. The retrospective design limits control over intraoperative medication adjustments and inter-provider practice variability. There was no control group for compression, further limiting the validity of the results. The relatively small sample size (n = 51) along with a single-center study may have reduced the statistical power, particularly for subgroup analyses. In addition, our study focused solely on the PACU period; relying on maximum pain score alone, time to discharge data were inconsistently recorded and could not be reliably analyzed; we did not assess post-discharge analgesia requirements or long-term outcomes. Future research should prioritize prospective, randomized controlled trials to validate our findings, explore optimal dosing strategies, and assess long-term outcomes such as patient satisfaction, cost-effectiveness, and quality of recovery. Integrating patient-reported outcomes and caregiver satisfaction would also enrich the understanding of dexmedetomidine’s role in pediatric anesthesia.

## Conclusions

This retrospective case series suggests that dexmedetomidine may offer a feasible opioid-sparing approach to postoperative analgesia in pediatric adenotonsillectomy. However, due to the small sample size and lack of a control group, conclusions about efficacy should be interpreted with caution. Moreover, No patients encountered intraoperative or postoperative bradycardia or hypotension necessitating pharmaceutical intervention. PACU and anesthesia documentation did not indicate any hemodynamic instability. The lack of postponed recovery may reinforces the safety of dexmedetomidine in our group of patients.
